# Ictal direct current shifts contribute to defining the core ictal focus in epilepsy surgery

**DOI:** 10.1093/braincomms/fcac222

**Published:** 2022-09-03

**Authors:** Mitsuyoshi Nakatani, Morito Inouchi, Masako Daifu-Kobayashi, Tomohiko Murai, Jumpei Togawa, Shunsuke Kajikawa, Katsuya Kobayashi, Takefumi Hitomi, Takeharu Kunieda, Satoka Hashimoto, Motoki Inaji, Hiroshi Shirozu, Kyoko Kanazawa, Masaki Iwasaki, Naotaka Usui, Yushi Inoue, Taketoshi Maehara, Akio Ikeda

**Affiliations:** Department of Neurology, Kyoto University Graduate School of Medicine, 54 Shogoin-Kawaharacho, Sakyo-ku, Kyoto 606-8507, Japan; Department of Epilepsy, Movement Disorders and Physiology, Kyoto University Graduate School of Medicine, 54 Shogoin-Kawaharacho, Sakyo-ku, Kyoto 606-8507, Japan; Department of Neurology, Kyoto City Hospital, 1-2 Mibuhigashitakadacho, Nakagyo-ku, Kyoto 604-8845, Japan; Department of Neurology, Kyoto University Graduate School of Medicine, 54 Shogoin-Kawaharacho, Sakyo-ku, Kyoto 606-8507, Japan; Department of Neurology, Kyoto University Graduate School of Medicine, 54 Shogoin-Kawaharacho, Sakyo-ku, Kyoto 606-8507, Japan; Department of Neurology, Kyoto University Graduate School of Medicine, 54 Shogoin-Kawaharacho, Sakyo-ku, Kyoto 606-8507, Japan; Department of Neurology, Kyoto University Graduate School of Medicine, 54 Shogoin-Kawaharacho, Sakyo-ku, Kyoto 606-8507, Japan; Department of Neurology, Kyoto University Graduate School of Medicine, 54 Shogoin-Kawaharacho, Sakyo-ku, Kyoto 606-8507, Japan; Department of Laboratory Medicine, Kyoto University, 54 Shogoin-Kawaharacho, Sakyo-ku, Kyoto 606-8507, Japan; Department of Neurosurgery, Ehime University Graduate School of Medicine, Shitsukawa, Toon City, Ehime 791-0295, Japan; Department of Neurosurgery, Kyoto University Graduate School of Medicine, 54 Shogoin-Kawaharacho, Sakyo-ku, Kyoto 606-8507, Japan; Department of Functional Neurosurgery, Tokyo Medical and Dental University, 1-5-45 Yushima, Bunkyo-ku, Tokyo 113-8510, Japan; Department of Functional Neurosurgery, Tokyo Medical and Dental University, 1-5-45 Yushima, Bunkyo-ku, Tokyo 113-8510, Japan; Department of Neurosurgery, Nishi-Niigata Chuo National Hospital, 1-14-1 Masago, Nishi-ku, Niigata 950-2085, Japan; Department of Neurology, National Center Hospital, National Center of Neurology and Psychiatry, 4-1-1 Ogawa-higashi-cho, Kodaira-shi, Tokyo 187-8551, Japan; Department of Neurosurgery, National Center Hospital, National Center of Neurology and Psychiatry, 4-1-1 Ogawa-higashi-cho, Kodaira-shi, Tokyo 187-8551, Japan; Department of Neurosurgery, Shizuoka Institute of Epilepsy and Neurological Disorders, Urushiyama 886, Aoi-ku, Shizuoka 420-8688, Japan; Department of Psychiatry, Shizuoka Institute of Epilepsy and Neurological Disorders, Urushiyama 886, Aoi-ku, Shizuoka 420-8688, Japan; Department of Functional Neurosurgery, Tokyo Medical and Dental University, 1-5-45 Yushima, Bunkyo-ku, Tokyo 113-8510, Japan; Department of Epilepsy, Movement Disorders and Physiology, Kyoto University Graduate School of Medicine, 54 Shogoin-Kawaharacho, Sakyo-ku, Kyoto 606-8507, Japan

**Keywords:** intractable focal epilepsy, presurgical evaluation, ictal direct-current (DC) shifts, ictal high-frequency oscillations (HFOs), surgical outcome

## Abstract

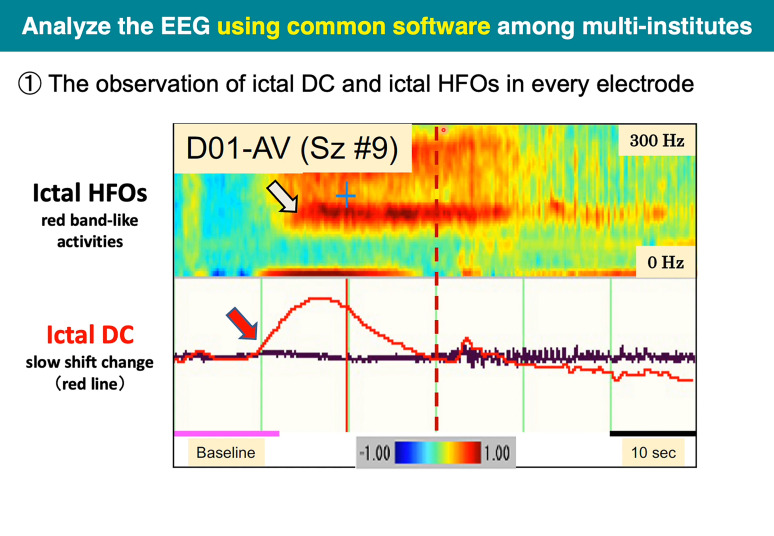
 Identifying the minimal and optimal epileptogenic area to resect and cure is the goal of epilepsy surgery. To achieve this, EEG analysis is recognized as the most direct way to detect epileptogenic lesions from spatiotemporal perspectives. Although ictal direct-current shifts (below 1 Hz) and ictal high-frequency oscillations (above 80 Hz) have received increasing attention as good indicators that can add more specific information to the conventionally defined seizure-onset zone, large cohort studies on postoperative outcomes are still lacking. This work aimed to clarify whether this additional information, particularly ictal direct-current shifts which is assumed to reflect extracellular potassium concentration, really improve postoperative outcomes. To assess the usefulness in epilepsy surgery, we collected unique EEG data sets recorded with a longer time constant of 10 s using an alternate current amplifier. Sixty-one patients (15 with mesial temporal lobe epilepsy and 46 with neocortical epilepsy) who had undergone invasive presurgical evaluation for medically refractory seizures at five institutes in Japan were retrospectively enrolled in this study. Among intracranially implanted electrodes, the two core electrodes of both ictal direct-current shifts and ictal high-frequency oscillations were independently identified by board-certified clinicians based on unified methods. The occurrence patterns, such as their onset time, duration, and amplitude (power) were evaluated to extract the features of both ictal direct-current shifts and ictal high-frequency oscillations. Additionally, we examined whether the resection ratio of the core electrodes of ictal direct-current shifts and ictal high-frequency oscillations independently correlated with favourable outcomes. A total of 53 patients with 327 seizures were analyzed for wide-band EEG analysis, and 49 patients were analyzed for outcome analysis. Ictal direct-current shifts were detected in the seizure-onset zone more frequently than ictal high-frequency oscillations among both patients (92% versus 71%) and seizures (86% versus 62%). Additionally, ictal direct-current shifts significantly preceded ictal high-frequency oscillations in patients exhibiting both biomarkers, and ictal direct-current shifts occurred more frequently in neocortical epilepsy patients than in mesial temporal lobe epilepsy patients. Finally, although a low corresponding rate was observed for ictal direct-current shifts and ictal high-frequency oscillations (39%) at the electrode level, complete resection of the core area of ictal direct-current shifts significantly correlated with favourable outcomes, similar to ictal high-frequency oscillation outcomes. Our results provide a proof of concept that the independent significance of ictal direct-current shifts from ictal high-frequency oscillations should be considered as reliable biomarkers to achieve favourable outcomes in epilepsy surgery. Moreover, the different distribution of the core areas of ictal direct-current shifts and ictal high-frequency oscillations may provide new insights into the underlying mechanisms of epilepsy, in which not only neurons but also glial cells may be actively involved via extracellular potassium levels.

## Teaser

Nakatani *et al.* collected large human EEG data sets from multiple institutions to assess the utility of ictal direct-current shifts in epilepsy surgery. Complete resection of the core region showing ictal direct-current shifts, commonly observed in neocortical epilepsy, independently correlated with favourable seizure outcomes, as well as ictal high-frequency oscillations.

## Graphical Abstract

**Graphical Abstract:**
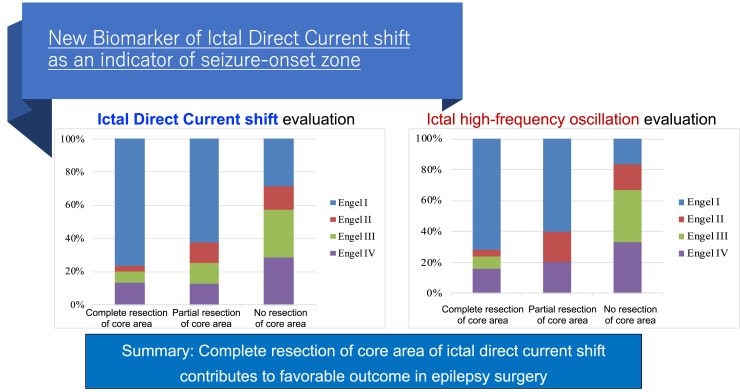

## Introduction

Epilepsy is a common neurological disorder characterized primarily by iterative seizures derived from paroxysmal, excessive neuronal activities. In drug-resistant focal epilepsy patients, epilepsy surgery is a promising treatment choice, but identifying a sufficient and specific seizure-onset zone (SOZ) is essential to achieve a favourable surgical outcome. Although it is challenging to delineate the ‘epileptogenic zone’ that needs to resect for the complete abolition of seizures,^1^ we should approach this goal using various methodologies. Advances in digital EEG techniques have provided an extremely wide range of brain activities from slow potential activities less than 1 Hz and much slower, such as infraslow activity (ISA)^2,3^ and ictal direct-current shifts (icDCs),^4,5^ to high-frequency oscillations (HFOs) greater than 80, 300 Hz and more.^6^ Little attention has been given to ictal slow potentials in recent decades. Examination of HFOs, which reflect the hyperexcitability of neurons,^7^ provides direct knowledge applicable to investigating the epileptogenic zone, but the situation in which one-third of epileptic patients are intractable remains unchanged. The accumulated observation of ISA in animals^8^ and humans^9^ has revealed that ISA modulates cortical excitability^2,10^ and influences seizure susceptibility.^3^ icDCs at seizure onset, a type of ISA, were first reported in experimental animal studies of an acute seizure model using a DC amplifier in the 1960s.^11^ As a result of the subsequent accumulated research data, we obtained the following findings: (i) icDCs were recorded in focal epilepsy patients with a longer time constant (TC) of 10 s (i.e. the high-pass filter of 0.016 Hz) using an alternate current (AC) amplifier instead of a DC amplifier^12,13^; (ii) ∼90% of icDCs were observed using subdural electrodes, and ∼34% were observed by scalp EEG analysis in patients with epilepsy^14^; (iii) icDCs were detected in a more restrictive area than the conventionally defined seizure-onset zone (cSOZ) and earlier than icHFOs during seizures at the individual level.^5,15^ These findings suggest that icDCs can represent the core epileptogenic area,^12,13^ and subsequent studies have verified and validated the importance of icDCs.^16,17^ However, most of these reports are derived from single-centre data, and comprehensive studies using multi-institute data have not yet been conducted, presumably because no standardized common metrics are available to identify icDCs. Importantly, a further challenge that must be overcome is to ensure the significance of icDCs in predicting better outcomes after epilepsy surgery because only a few reports have documented the association between icDCs and postoperative outcomes.^4,18^ Therefore, in this study, we aimed to clarify the occurrence pattern of icDCs using multi-institute data based on our previously reported unified method^19^ and to elucidate whether resection of the core area of icDCs correlates with favourable outcomes.

## Methods

### Data acquisition

**Figure 1:**
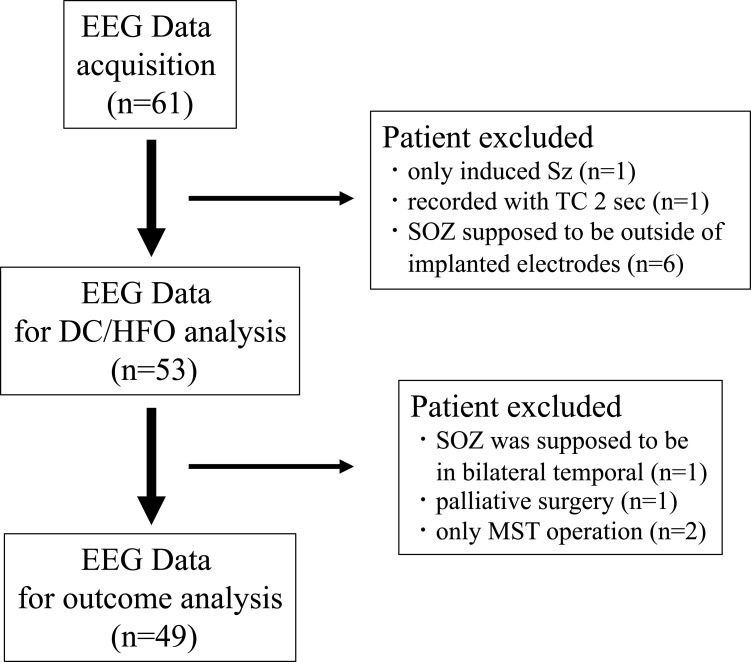
Inclusion criteria for the EEG analysis of icDCs/icHFOs and postoperative outcome analysis.

As a retrospective cohort study, we enrolled intractable focal epilepsy patients who had undergone invasive presurgical evaluation. Forty-five patients were included from five institutes in Japan (Kyoto University; Tokyo Medical and Dental University; National Center Hospital, National Center of Neurology and Psychiatry; Nishi-Niigata Chuo National Hospital; and Shizuoka Institute of Epilepsy and Neurological Disorders) from 2015 to 2017 (The data of 16 previously reported patients^5^ were also added). A total of 61 patients were finally included in this study (Fig. 1). The results of clinical examinations such as MRI and FDG-PET, the intracranial location of the implanted electrodes, the pathology of extracted specimens labelled by Palmini’s classification,^20^ and the surgical outcomes were recorded. The research protocol in this study was approved by the Ethical Committee of Kyoto University Graduate School of Medicine (IRB R0600), and mutual data sharing and analysis among institutes were also validated by each institute’s ethical committee. Written informed consent was also acquired from all the patients before presurgical evaluation.

### Invasive EEG recording and inclusion versus exclusion criteria of seizures

The EEG data were recorded using an EEG 1100/1200 system (Nihon Kohden, Tokyo, Japan) with a sampling rate of 1000 Hz/2000 Hz and a high-pass filter of 0.016 Hz equal to a TC of 10 s. The implanted subdural electrodes were disks of 3 mm diameter made of platinum (Ad-Tech, Racine, WI, USA; Unique Medical Co., Ltd, Tokyo, Japan), and depth electrodes were also implanted to explore the deeper areas in 19 patients. Both system reference and ground electrodes were set outside the cranium, such as the mastoid process and subgaleal area.

The inclusion criteria to analyze seizures were the same as those in our previous study.^5^ Briefly, we adopted spontaneous clinical seizures with habitual ictal semiology and subclinical seizures with identical conventional ictal EEG changes to clinical seizures. The number of included seizures ranged from two to a maximum of ten in each patient. The exclusion criteria of the analysis are described in Fig. 1. For EEG analysis, we excluded patients who showed seizures induced only by electric cortical stimulation (*n* = 1), whose data were recorded with a TC of 2 s (*n* = 1), and whose SOZ was uncovered by implanted electrodes (*n* = 6). Overall, 53 patients with 327 seizures met the inclusion criteria for EEG analysis. In the postoperative outcome analysis, we excluded patients whose SOZ was presumed bilateral temporal (*n* = 1) and who had only undergone palliative surgery (*n* = 1) or multiple subpial transection operations (*n* = 2); thus, 49 patients were ultimately included. Because this study is a retrospective analysis of icDCs and icHFOs, the actual surgeries were performed at each institute primarily based on the results of clinical examinations, including conventional ictal EEG and interictal HFOs, which have already been established as good indicators of epileptogenicity to a certain level.

### Analysis methods

We used a custom-made script written in MATLAB (The MathWorks, Natick, MA, USA) for the patients in a previous study^5^ and patients 1–9, and wide-band EEG analysis software (Nihon Kohden, Tokyo, Japan) for patients 10–45. Only icDCs and icHFOs that were both reproducible in terms of location, the occurrence pattern of the polarity of icDCs and the same band-like activities of icHFOs were considered for subsequent analysis. Like conventional EEG analysis, the electrode that is not involved during seizures must be appropriately selected as the reference electrode to highlight the core area of icDCs and icHFOs. The electrode placed on the mastoid was first used as the reference electrode to display the EEG data. If it failed or contained some artefacts, to minimize artefacts, the display reference electrode was correctly altered to the electrode most distant from the suspected SOZ^13^ or the averaged potential of all intracranial electrodes as in our previous study.^5,19^ The display condition was maintained in each individual analysis.

**Figure 2:**
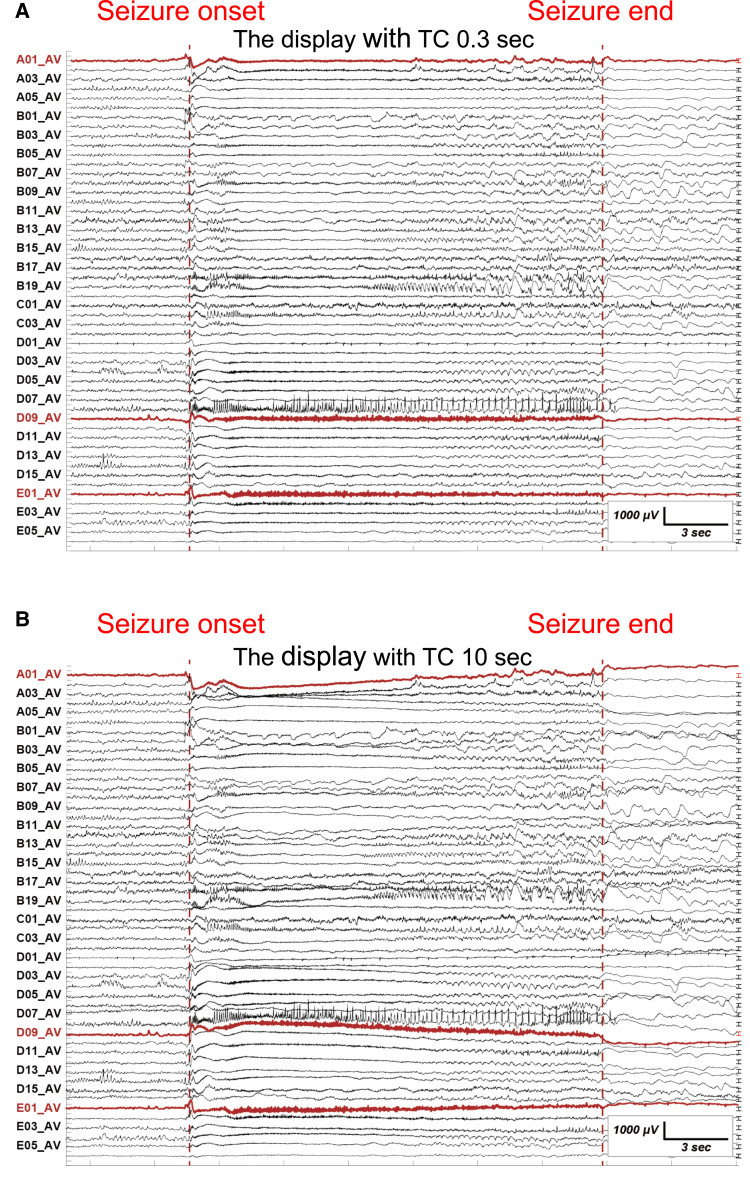
**The detection of the two core electrodes of icDCs and icHFOs.** (**A**), (**B**) The representative ictal seizure of a left MTLE patient (Patient 10 in Supplementary Table 1) is shown with the display setting with a TC of 0.3 s (**A**) and a TC of 10 s (**B**). Each line demonstrates the EEG activity of each electrode (from A01 to E05, part of the implanted electrodes) with reference to the average montage of all implanted electrodes. The (bold) red dotted vertical line indicates the seizure onset and seizure end determined by conventional EEG analysis. The two electrodes A01 and D09 in (bold) red colour are identified as the core electrodes of icDCs based on the priority of the selection criteria. In a same way, D09 and E01 in (bold) red colour are identified as the core electrodes of icHFOs.

Because the analysis methods of icDCs and icHFOs have not been established to be common among institutes, we adopted the common metrics we proposed previously^5,19^ to analyze the EEG data in this study. First, conventional ictal EEG activity in the general setting of a TC of 0.3 s was used to detect seizure onset. The detected seizure-onset time was set as ‘time 0’ to evaluate the latency difference of icDCs and icHFOs (Fig. 2). icDCs were defined as sustained negative and/or positive potentials longer than 3 s, viewed in a setting of a TC of 10 s and a 60 s time window with ‘time 0’ in the middle.^13^ icHFOs were defined as discrete and sustained ‘band-like’ power increases above 80 Hz after short-term Fourier transformation (STFT) analysis in a 100 ms epoch corresponding to a frequency resolution of 10 Hz at each epoch with half-width overlapping, viewed in a setting of a 30 s time window with ‘time 0’ in the middle.^5,15^ The analysis time window was set up separately so that each parameter of icDCs and icHFOs could be easily recognized through visual inspection.

**Figure 3:**
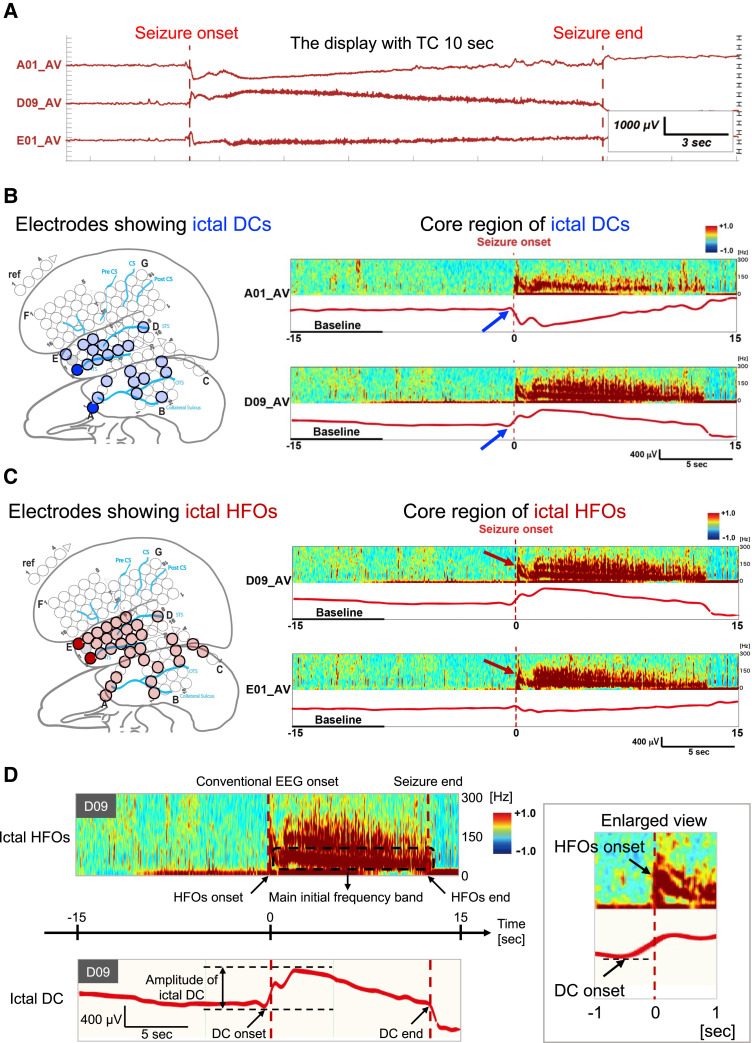
**The analysis of the two core electrodes of icDCs and icHFOs.** (**A**) This figure is an enlarged view of the three electrodes identified as the core electrodes of icDCs and icHFOs (A01 and D09 for icDCs, D09 and E01 for icHFOs, also refer to Fig. 2). Note that both positive and negative polarity of icDCs were observed at seizure onset within a seizure. (**B**), (**C**) These figures illustrate the results of the determination of the two core electrodes of icDCs (**B**) and icHFOs (**C**) among the implanted electrodes in the same patient as Fig. 2. The configuration of the implanted electrodes during presurgical evaluation is shown in the brain schema (left part). Dark blue circles indicate the electrodes that showed icDCs at seizure onset, whereas dark red circles indicate the electrodes that showed icHFOs at seizure onset. The electrodes that reproducibly met the priority of the selection criteria among the collected seizures were identified as the two core electrodes of icDCs and icHFOs (see the Methods section). For example, the A01 and D09 electrodes (dark blue circle) were identified as core electrodes of icDCs, whereas the D09 and E01 electrodes were identified as those of icHFOs (dark red circle) in this representative patient. The figures in right panel feature the activities of the two identified core electrodes of icDCs and icHFOs, each independently. The (bold) red dotted vertical line is the seizure onset based on conventional EEG activity. Each column in the right panel contains the power spectrum (the upper section in each column, 0–300 Hz) and the activity with a 1 Hz LPF (lower section in each column) with a 60 s time window. (**D**) This figure describes the evaluation factors in the two core electrodes of icDCs and icHFOs, and each is described independently. The onset time, duration, and amplitude of icDCs were evaluated in the core electrodes of icDCs. For icHFOs, we also estimated the onset time, duration, and maximum initial power spectrum band range. We set the fundamental band-like activities as their main frequency (see the Methods section).

Second, because both positive and negative pairwise icDCs can occur at the onset of one seizure^12,13^ regardless of the position of the reference electrode, the two most prominent electrodes among implanted electrodes were independently defined as the core electrodes of icDCs and icHFOs. The priority of the selection was the following order: the earliest, longest and largest amplitude/power spectrum. The representative results of the two core electrodes in one patient are described in Fig. 2A, B and Fig. 3A–C.

Third, we evaluated the following factors in these two core electrodes (Fig. 3D). The onset time, duration and amplitude (from onset to peak) of icDCs were measured. In the analysis of the newly collected patients, the analysis time window was fixed because of the limitations of the software. Thus, the mean duration of icDCs was calculated only for the patients showing icDCs that returned to baseline within 30 s after ‘time 0’ (17/39 patients). For icHFOs, we estimated the onset time, duration, and initial power spectrum band. When different bands of icHFOs (fundamental, second harmonic, and third harmonic activities) were present within a seizure,^15^ we regarded fundamental band-like activities, namely the lowest band activities among several bands, as their main frequency. In all steps, the baseline was set as the first 20% of the time window in each seizure (see more detail^5,19^).

At least two board-certified neurologists/neurosurgeons individually identified the two core electrodes of both icDCs and icHFOs and assessed the evaluation factors mentioned above separately. If a disagreement occurred, it was resolved through discussion.

### Outcome evaluation

The extent of surgical resection was decided entirely independently of this study by the neurologists/neurosurgeons at each institute based on all the available clinical information. The surgical outcome of each patient was rated by the Engel classification,^21^ at least for the 1-year follow-up after surgery. Engel class I was regarded as a good outcome, whereas Engel classes II, III, and IV were regarded as poor outcomes. Preoperative and postoperative MRI are commonly used to outline the extent of actual surgical resection by neurosurgeons at each institute. Next, we evaluated how the resection of the core area of icDCs and icHFOs correlated with the postoperative outcome.

### Statistical analysis

To compare the latency of icDCs and icHFOs from ‘time 0’, we used the Wilcoxon rank-sum test to evaluate the patients who showed either parameter and used Wilcoxon’s signed-rank test to assess those who showed both parameters. The Wilcoxon rank-sum test was also used to compare their onset times in each patient. A χ^2^ test was used to evaluate the relationships between the outcome and degree of complete resection. Logistic regression analysis was used to obtain the receiver operating characteristic (ROC) curve for favourable outcomes. A significant value was set at *P* < 0.05 for all analyses. All statistical analyses were performed using JMP software (JMP Pro version 13; SAS Institute, Cary, NC).

### Data availability

The anonymized raw EEG data sets of the participants in this study are available on the secured online data repository at Kyoto University. All the data are available from the corresponding author on reasonable request.

## Results

A summary of the enrolled patients is shown in Table 1. Among the 61 patients registered, 15 had mesial temporal lobe epilepsy (MTLE), and 46 had neocortical epilepsy (NE). Frontal lobe epilepsy and lateral temporal lobe epilepsy accounted for most of the forms of epilepsy in the NE group. No significant difference was found in sex, age at onset, or age at surgery between the MTLE and NE groups. The details of the clinical information of the 45 newly enrolled patients are described in Supplementary Table 1 (the details of the 16 previously enrolled patients have been described previously^5^).

**Table 1:** Demographics of patients in this study

	Mesial temporal epilepsy (MTLE)	Neocortical epilepsy (NE)
Patients # (*n* = 61)	15	46
Gender		
Male	7	30
Female	8	16
Age at onset		
(mean ± SD)	12.0 ± 7.0	
(3–27)	11.7 ± 9.8	
(0–43)		
Age of surgery		
(mean ± SD)	31.5 ± 10.9	
(11–52)	27.8 ± 12.2	
(12–61)		
Lesion		
Frontal	0	25
Temporal	15 (mesial)	11 (lateral)
Parietal	0	4
Occipital	0	4
fronto-parietal	0	1
templo-parietal	0	1

### General features of icDCs and icHFOs

**Figure 4:**
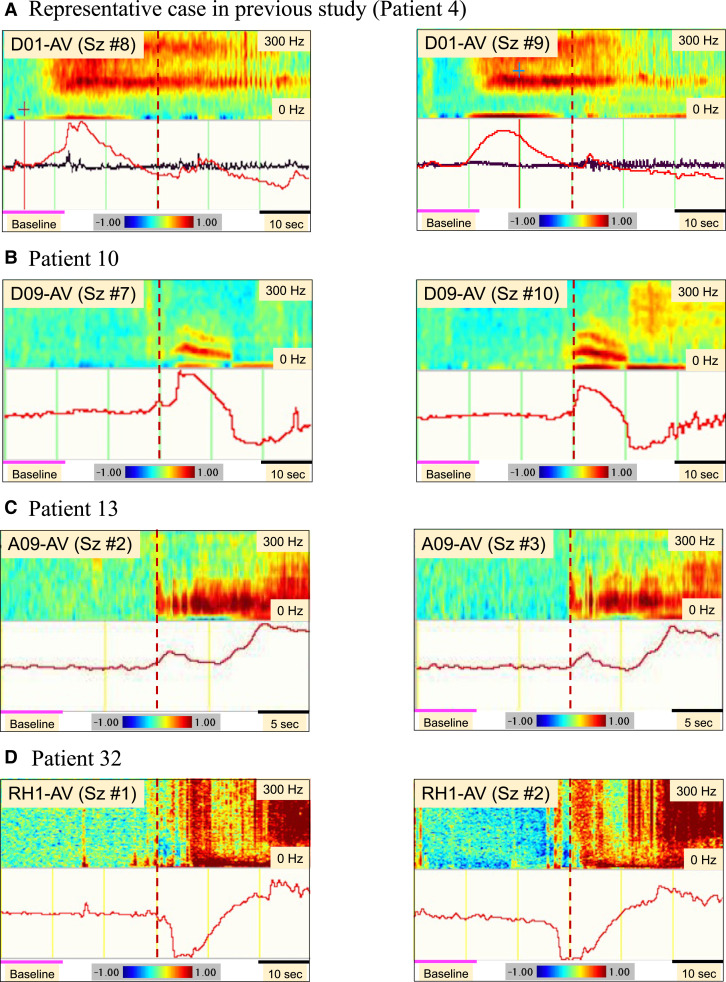
**Various types of icDCs and icHFOs that appear reproducibly in each patient.** We illustrated several occurrence patterns of icDCs and icHFOs depending on the patients. One patient from our previous report, Patient 10, Patient. 13 and Patient 32 from different institutes are picked up to show the reproducibility of icDCs and icHFOs in different seizures (**B**: Sz #7 and #10 in Pt. 10; **C**: Sz #2 and #3 in Pt. 13; **D**: Sz #1 and #2 in Pt. 32). In each column, the defined core electrode of icDCs was described with STFT analysis in a 100 ms epoch of high-frequency oscillations. The (bold) red dotted vertical line indicates the seizure onset identified by conventional EEG change. The bold pink line (from 0 to 12 s) was set as the baseline, which corresponds to the first 20% of the analysis time window. In every patient, here, averaged montage was selected as the reference electrode to display the EEG change during seizures. Note that patient-specific patterns of icDCs and icHFOs appearance can exist depending on the patient. (**A**) Note that both icDCs and icHFOs preceded several 10 s before the conventional EEG onset. (**B**) The negative polarity (upward) of icDCs was reproducibly observed before or at seizure onset, accompanied by a decremental pattern of icHFOs. (**C**) The two humped icDCs were repeatedly observed in different seizures. In this patient, repetitive spikes were prominent during seizures; thus, clear ictal band-like HFOs were not identified in this electrode. (**D**) Importantly, positive polarity (downward) of icDCs can sometimes be observed, as in the case of this patient. Obvious ictal band-like HFOs were not detected in this electrode.

Fifty-three of 61 patients were fully examined by EEG analysis. The representative examples of icDCs and icHFOs were described in Fig. 4. We verified that the occurrence patterns of icDCs and icHFOs, including their polarity, morphology, and duration, varied from patient to patient, but are reproducibly observed in a patient-specific way even in different seizures. The results of each evaluation factor of icDCs and icHFOs are described in Table 2 with reference to previous reports.^4,5,13,17,18,22^ The occurrence rates of icDCs and icHFOs among patients were 92 and 71%, respectively, whereas those among seizures were 86 and 62%, respectively. The higher occurrence rate of icDCs aligned with previous study findings.^4,5^ Although both icDCs and icHFOs suggested an epileptic focus, the concordance rate of the core regions of icDCs and icHFOs was unexpectedly only 39% when evaluated at the electrode level. From the patient perspective, only 6 of 53 patients showed 100% correspondence between the core regions of icDCs and icHFOs. Representative examples of the different concordance rates (0, 50, or 100%) are illustrated in Supplementary Fig. 1. The mean amplitude of icDCs was 1037 ± 570 μV [min: 353·8 μV (Pt. 33), max: 2876 μV (Pt. 32)]. The icDC duration was ∼15.8 ± 7.8 s on average [min: 3 s (Pt. 11, 35), max: 27 s (Pt. 22)]. icHFOs were commonly observed, but temporal synchronized patterns, such as incremental, decremental, and stationary patterns, varied among patients.^19^ Although several bands of icHFOs were observed, their fundamental frequency showed ripple range activity, as in previous studies.^23^ We also found that the duration of icHFOs was shorter (∼7.0 ± 4.1 s [min: 1 s (Pt. 11, 35), max: 13 s (Pt. 21)]) than that of icDCs under the same methodological conditions of the software used in the present study.

**Table 2:** The comparison with previous reports discussing ictal DC and ictal HFOs

	Amplifier	Occurrence rate among patients (%)		Occurrence rate among seizures (%)		Correspondence of core electrodes of ictal DC and HFOs (%)	ictal DC amplitude (μV)	ictal DC duration (s)	ictal HFOs frequency (Hz)	ictal HFOs duration (s)
		ictal DCs	ictal HFOs	ictal DCs	ictal HFOs					
Nakatani et al.^19^ (*n* = 61)	AC	92	71	86	62	39	1037 ± 570	15.8 ± 7.8^a^	R (FR)	7.0 ± 4.1^a^
Ikeda et al.^13^ (*n* = 9)	AC	82 (subdural) 84 (scalp)	–	85 (subdural) 23 (scalp)	–	–	200—(subdural) 50—(scalp)	–	–	–
Modur and Scherg^18^ (*n* = 1)	AC	100	100	100	75	10–75 (no detail)	–	−25	R	Sustained (no detail)
Kim et al.^22^ (*n* = 11)	DC	91	–	69.5	–	–	800−10 000	1−493	–	–
Wu et al.^4^ (*n* = 15)	AC	100	67	91	81	19.3	1700 ± 910	5 − 180	R, FR	–
Kanazawa et al.^5^ (*n* = 16)	AC	75	50	71.3	46.3	–	903.1 ± 462.8	35.5 ± 15.6	R, FR	10.7 ± 9.7
Thompson et al.^17^ (*n* = 15)	AC	100	–	100	–	–	300−8500	− over 100	–	–

DC, direct-current, AC, alternate current; ictal DCs, ictal direct-current shifts; ictal HFOs, ictal high-frequency oscillations.

^a^ Long-lasting icDCs or icHFOs beyond 30 s analysis time-window after the seizure onset were excluded due to the limitation of the software.

### Comparison of the onset time between icDCs and icHFOs

**Figure 5:**
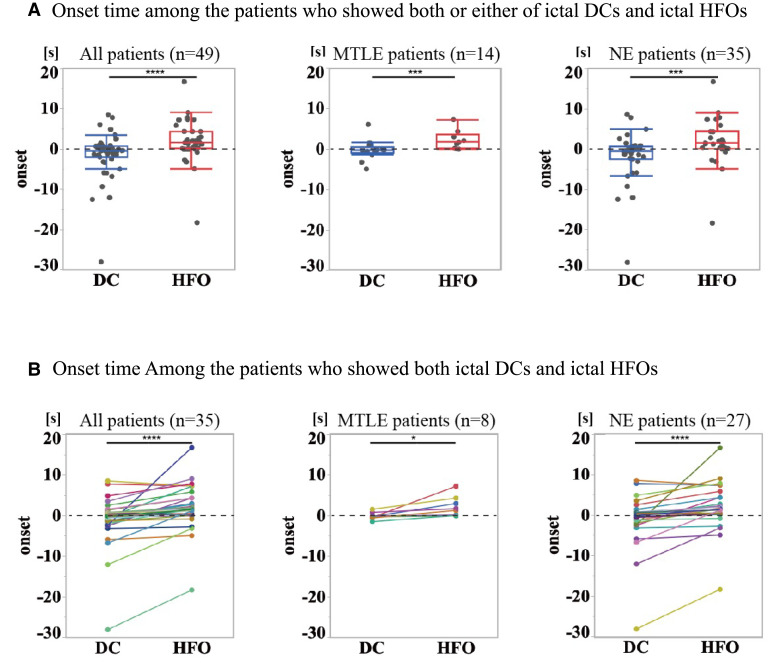
**Temporal relationships between the onset of icDCs and icHFOs.** (**A**), (**B**) Because the appearance rate of icDCs and icHFOs differed among patients, we compared their latencies in the following two ways. One was a comparison among patients who showed both or either icDCs and icHFOs at onset, and the other was a comparison among the patients who showed both icDCs and icHFOs at onset. (**A**) Among 53 patients, 49 showed both or either icDCs or icHFOs. The black dotted line indicates the seizure-onset time determined by conventional ictal EEG. The averaged latency of icDCs and icHFOs compared with conventional ictal EEG onset among all seizures was calculated for each patient. Each dot represents the averaged latency of icDCs or icHFOs in one patient. icDCs significantly preceded icHFOs among all patients who showed either of these activities (−1.6 ± 5.6 s for icDCs, 1.9 ± 5.1 s for icHFOs; *P* < 0.001; Wilcoxon rank-sum test). After dividing the patients into two groups, MTLE and NE patients, a significant difference was observed between the latency of icDCs and icHFOs [MTLE group: −0.3 ± 2.4 s for icDCs, 2.2 ± 2.3 s for icHFOs (*P* < 0.01); NE group: −0.3 ± 2.8 s for icDCs, 1.7 ± 2.5 s for icHFOs (*P* < 0.01)]. (**B**) Among 53 patients, 35 showed both icDCs and icHFOs. The figure configuration is the same as in (**A**). The different coloured dots and lines represent the averaged latency of icDCs and icHFOs in each patient. A statistically significant difference was observed among all patients who showed both (−1.1 ± 5.8 s for icDCs, 1.9 ± 5.3 s for icHFOs; *P* < 0.001; Wilcoxon signed-rank test). Similar results were also obtained after dividing the patients into two groups: MTLE and NE patients [MTLE group: −0.1 ± 0.9 s for icDCs, 2.2 ± 2.4 s for icHFOs (*P* < 0.05); NE group: −1.3 ± 6.6 s for icDCs, 1.8 ± 5.8 s for icHFOs (*P* < 0.001)].

The averaged latency of icDCs and icHFOs from the conventional ictal EEG onset ‘time 0’ across all analyzed seizures is shown in Fig. 5A, B. Among the 53 patients in EEG analysis, 49 showed both or either of the two activities. The latency from ‘time 0’ was significantly earlier in icDCs than in icHFOs. After dividing the patients into MTLE (*n* = 14) and NE (*n* = 35) groups, a significant difference existed within both groups (Fig. 5A). We also compared the latency among 35 patients who showed both icDCs and icHFOs. Similarly, a significant difference was found between their latencies, with icDCs occurring earlier than icHFOs. A significant difference was also observed in both the MTLE (*n* = 8) and NE (*n* = 27) groups (Fig. 5B).

**Figure 6:**
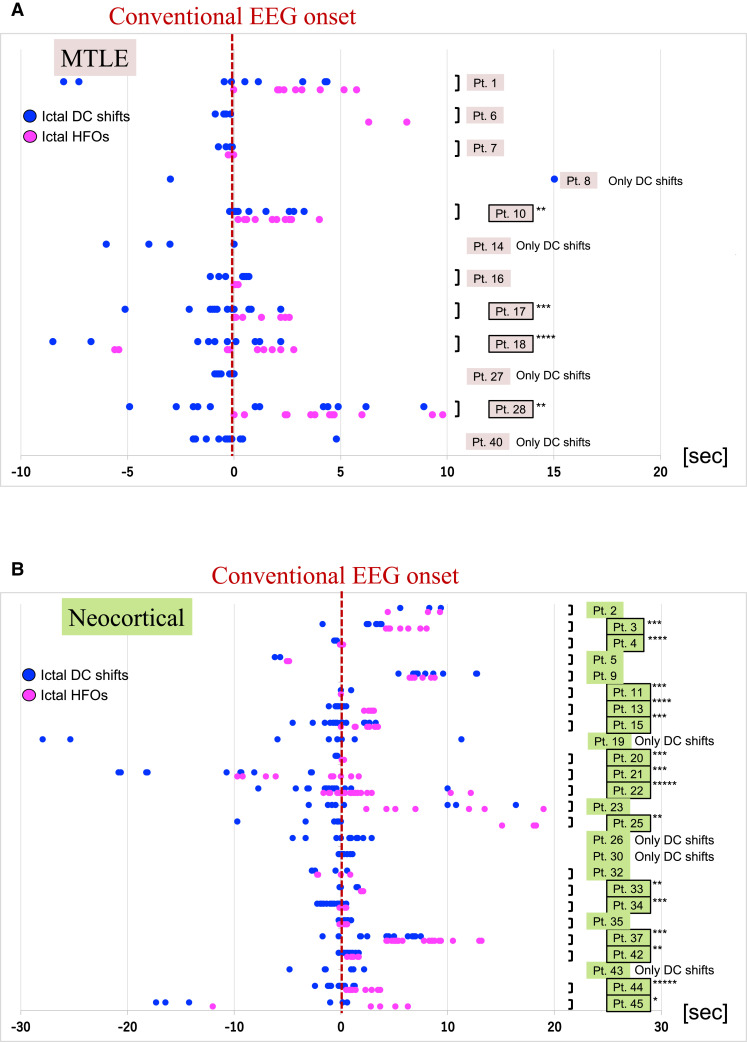
**The difference of the onset of icDCs and icHFOs between MTLE and NE patients.** (**A**), (**B**) We plotted the onset time of icDCs and icHFOs in the MTLE group (**A**) and in the NE group (**B**). Dark blue dots represent the onset time of icDCs in each seizure, whereas light magenta dots represent that of icHFOs in each seizure. The (bold) red dotted vertical line is the conventional EEG onset in each seizure. Four patients showed that icDCs significantly preceded icHFOs among 12 newly recruited MTLE patients. Four patients (patients 8, 14, 27 and 40) with icDCs only and three patients (patients 6, 7 and 16) with a small number of icHFOs were not suitable for statistical analysis. Among 25 NE patients among the newly recruited patients, icDCs significantly preceded icHFOs in 15 patients except in four patients (patients 19, 26, 30 and 43) with only icDCs and three patients (patients 5, 32 and 35) with an insufficient number of seizures. Three patients (patients 2, 9 and 23) showed no significant difference between their onset times. Statistical differences are described using asterisks as follows (*: *P* < 0.05, **: *P* < 0.03, ***: *P* < 0.01, ****: *P* < 0.001, *****: *P* < 0.0001).

We also compared the latency of the two activities in each of 37 patients (the details of the remaining 16 of 61 patients have been described previously^5^). Among the 12 MTLE patients of 37 patients (Fig. 6A), icDCs significantly preceded icHFOs in four patients. Only Patient 1 showed no significant difference. Seven patients were not suitable for statistical analysis [four without icHFOs (Pt. 8, 14, 27, 40) and three with an insufficient number of seizures (Pt. 6, 7, 16)]. Among the 25 NE patients of 37 patients (Fig. 6B), icDCs significantly preceded icHFOs in 15 patients. Three patients showed no significant difference. Seven patients were not statistically applicable [four without icDCs (Pt. 19, 26, 30, 43) and three with an insufficient number of seizures (Pt. 5, 32, 35)]. We concluded that icDCs significantly preceded icHFOs in both MTLE and NE patients among and within patients; additionally, icDCs were more pronounced in NE patients.

### Relationships between the resection of the core areas and surgical outcomes

**Figure 7:**
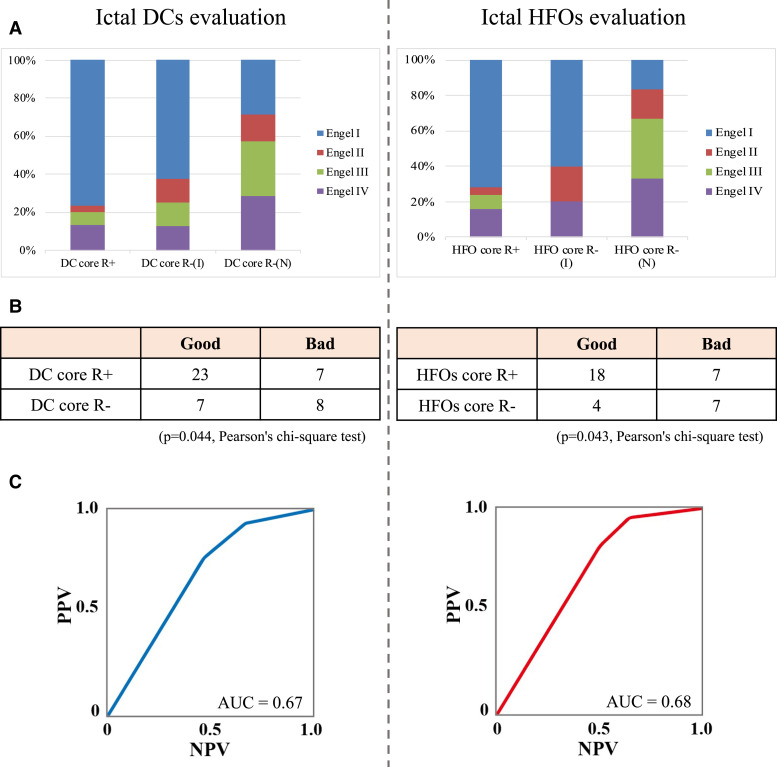
**Relationships between the postoperative outcome and extent of resection of the two core electrodes of icDCs or icHFOs.** The results based on icDCs are shown in the left part of the figure, and those based on icHFOs are shown in the right part of the figure. (**A**) The comparisons between the resection extent and the surgical outcome were assessed after dividing the patients into three groups: a complete resection group of the two core electrodes (DC core R+), an incomplete resection group of the two core electrodes (DC core R-(I)), and a non-resection group of the two core electrodes (DC core R-(N)). The same description is also used in HFOs analysis. The Engel classification was employed to evaluate the surgical outcome. Engel class I was regarded as a good outcome, whereas Engel classes II, III and IV were regarded as poor outcomes. The results obtained from the evaluation of icDCs and icHFOs indicate that favourable outcomes (Engel I) are predominantly related to the extent of resection of the two core electrodes. (**B**) In both icDCs and icHFOs, the complete resection of at least the two core electrodes was significantly independently associated with a good postoperative outcome (*P* = 0.044 (icDCs), *P* = 0.043 (icHFOs), Pearson’s χ^2^ test). (**C**) We assessed the diagnostic ability of each biomarker for the prediction of the outcome using a receiver operating characteristic (ROC) curve. The ROC curve for good postoperative prognosis is shown based on the positive predictive value (PPV) and negative predictive value (NPV) considering the ratio of the resection of the two core electrodes of icDCs and icHFOs, each shown independently. The area under the curve (AUC) values were 0.67 (icDCs) and 0.68 (icHFOs).

The relationships between surgical outcomes and the resection degree of the core areas are presented in Fig. 7. The mean period of follow-up after surgery was 22.1 ± 10 months. Of 49 patients considered in the outcome analysis, icDCs were detected in 45, and icHFOs were detected in 36. Of 45 patients who showed icDCs, 30 (66.7%) achieved favourable outcomes. Of 36 patients who showed icHFOs, 22 (61.1%) achieved favourable outcomes. Furthermore, of 30 patients who had complete resection of the core areas of icDCs, 23 (76.7%) achieved favourable outcomes (Fig. 7A, B, left). Similarly, of 25 patients who had complete resection of the core areas of icHFOs, 18 (72%) achieved favourable outcomes (Fig. 7A, B, right). Complete resection of the core areas of the two activities was significantly independently associated with favourable postoperative outcomes (Fig. 7B). Additionally, their ROC curve for better outcomes indicated that the area under the curve (AUC) values were 0.67 (icDCs) and 0.68 (icHFOs) (Fig. 7C). Thus, complete resection of the core areas of icDCs shows better than moderate predictive ability for favourable outcomes, similar to icHFOs.

### Relationships between the pathology and occurrence rate of icDCs/icHFOs

We described the relationships between the occurrence rate of icDCs/icHFOs and surgical outcome in each pathology^20^ (Table 3). Of 49 patients in the outcome analysis, 16 had focal cortical dysplasia (FCD) Type II, 11 had FCD Type I, five had hippocampal sclerosis (HS) and 17 had other pathologies. The occurrence rate of icDCs was higher than that of icHFOs in all pathologies. The highest occurrence rate of icDCs was observed in FCD Type IIb (99%) patients, but no clear difference was observed in the occurrence rate of icDCs among each pathology. The occurrence rate of icHFOs in FCD Type Ib and Type IIa patients was <50% compared with that in patients with other pathologies. Favourable outcomes were obtained for 100% of patients (5/5 patients) with HS, 72% (8/11 patients) with FCD Type I, 50% (8/16 patients) with FCD Type II and 71% (12/17 patients) with other pathologies. Although our cohort studies had few patients in each pathology, we concluded that a higher occurrence rate of icDCs than icHFOs was observed across all pathologies.

**Table 3:** Details of the occurrence rates of ictal DCs/ictal HFOs and surgical outcomes for each pathology

	HS^a^ (*n* = 5)	FCD Type I (*n* = 11)		FCD Type II (*n* = 16)		Others (*n* = 17)	Total (*n* = 49)
		Type IA (*n* = 8)	Type IB (*n* = 3)	Type IIA (*n* = 9)	Type IIB (*n* = 7)		
Occurrence rate (%)							
Ictal DCs	78	86	82	77	99	87	86
Ictal HFOs	63	86	38	49	83	58	65
Surgical outcome							
Engel I	5	5	3	3	5	12	32
Engel II	0	0	0	1	0	3	4
Engel III	0	2	0	2	1	0	5
Engel IV	0	1	0	3	1	2	10

HS, hippocampal sclerosis; FCD, focal cortical dysplasia; ictal DCs, ictal direct-current shifts; ictal HFOs, ictal high-frequency oscillations.

^a^ Including the FCD IIIa pathology of Patient 8 and Patient 10 in new ILAE classification.

## Discussion

We confirmed that both icDCs and icHFOs appeared very early in seizure onset. The present study highlighted three important points. First, we verified that icDCs precede icHFOs by analyzing multi-institute data based on a common analysis method.^5,12,13^ Second, the earlier appearance of icDCs was more significant in NE patients than in MTLE patients. Third, complete resection of the core area of icDCs and icHFOs was significantly independently correlated with favourable outcomes. To our best knowledge, this multi-institutional study is the first to show that resection of the core area of icDCs significantly correlates with favourable surgical outcomes.

### icDCs at the onset of a seizure

icDCs were identified with a higher probability than icHFOs among both domains of patients (92%) and seizures at the individual level (86%), a finding that was consistent with previous study findings (Table 2).^4,5^ icDCs have received increased attention because they may play a pivotal role in seizure genesis, at least in chronic epilepsy.^24^ icDCs are employed as an important factor to classify seizure phenotypes in mathematical models of epilepsy in theoretical research.^25,26^ Notably, the complete concordance of the core electrodes of both icDCs and icHFOs was only 39% despite both activities being observed within the cSOZ.^5,12,13^ Thus, each activity can originate from different cellular mechanisms,^4^ which might be consistent with the idea that icDCs reflect glia-neuronal interactions,^27,28^ whereas icHFOs reflect bursts of ictal neuronal activities.^7^ As will be discussed later in the limitation section, it remains possible that this difference is due to technical difficulties in observing extremely slow and fast frequency activities at the same time. However, we consider that our recording method is currently the most effective way to capture both activities. In contrast to the previous hypotheses that changes in blood perfusion,^29^ pCO2,^30^ pH,^31^ and blood brain barrier permeability^30^ may cause slow oscillations, similar to icDCs, during seizures, icDCs were recently considered to mainly reflect the extracellular potassium (K^+^) levels^32,33^ associated with neuronal excitability. An increased extracellular K^+^ concentration produced seizure-like episodes with a baseline shift in experimental studies.^34^ Additionally, previous experimental studies have clearly shown that astrocytes can both spontaneously depolarize or oscillate slowly apart from adjacent neuron activity^35^ or passively^36^ depolarize and that the intracellular glial potential certainly massively reflects the local field potential in the seizure focus together with the neuron-related field potential component.^37,38^ Therefore, the most plausible reason why icDCs precede conventional EEG onset and icHFOs is that astrocytes can be further activated by excessive extracellular K^+^ to regulate neuronal activities in their original role of maintaining extracellular homeostasis. Then, once the K^+^ concentration exceeds a certain level beyond the processing capacity of astrocytes, disruption of the extracellular environment may occur, resulting in actual epileptic seizures. Although the origin of abnormal K^+^ accumulation toward seizure onset remains controversial, it can be explained by either the accumulation of K^+^ efflux by the preictal excitation of interneurons^33,38^ or glial dysfunction of K^+^ buffering system related to potassium channels such as Kir4.1^39,40^ and impaired gap junctions around epileptic neurons.^32^ The involvement of glial cells is supported by a recent report that Kir4.1 depletion occurs in the subiculum in MTLE patients.^41^ In addition, from the synaptic transmitter perspective, it has been documented that astrocytes can serve as essential mediators of neuronal excitation in epileptic seizures through Ca^2+^-induced glutamate,^24^ glucose, and lactate.^42^ Accordingly, recent works have revealed that the overexpression of astrocytic brain-derived neurotrophic factor (BDNF) exacerbates epileptic seizures, whereas BDNF deletion reduces the neuronal firing rate,^39^ suggesting that astrocyte malfunction can affect the initiation and propagation of epileptic seizures. Notably, prominent icDCs can be observed at the earliest stage during the development of epileptogenicity in animal models of epilepsy. This finding may also imply the early involvement of glial malfunction.^43–45^ Thus, both gain and loss of function in astrocytes through repeated seizure occurrence strongly correlate with the development of epileptogenicity, finally leading to chronic epileptic seizures.^46^ Collectively, we propose that icDCs, which can reflect the disruption of the extracellular environment, such as the K^+^ concentration, are very important phenomenological indicators not only in detecting SOZ but also in considering the mechanisms of the transition from the interictal to ictal period.

### Ictal band-like HFOs during the seizure

Based on our findings, icHFOs seem to follow icDCs and conventional EEG onset. One possible reason is that the ‘visible’ ictal band-like HFOs above 80 Hz featured in this study seem to require a certain degree of synchronized neuronal activities. This finding can be simply attributed to the delayed appearance of icHFOs. Interestingly, delayed-onset and sustained icHFOs are considered more important than transient early-onset HFOs to differentiate the core region from the penumbra region in epilepsy patients.^47^ The preictal activation of interneurons supposedly occurs before seizure onset to suppress the hyperactivation of principal neurons.^33^ However, it ultimately fails, presumably owing to depolarization block of interneurons^48^ or synaptic inhibition by other interneurons,^49^ and epileptic seizures occur with explosive depolarization of principal neurons toward icHFOs. Given that icDCs precede either icHFOs or conventional ictal EEG onset, we propose that not only inhibitory neurons but also astroglial functions of K^+^ buffering may concurrently play a crucial role to mitigate excitatory neural hyperactivation toward seizure ignition.

### Difference between mesial temporal lobe epilepsy and neocortical epilepsy

Even at the individual level, icDCs preceding icHFOs were observed more often in NE patients than in MTLE patients (Fig. 6A, B), similar to a previous report.^50^ The following factors should be considered possible reasons. First, because the implanted electrodes were not right over the hippocampus, the recorded activities might be those already propagated from the hippocampus. The second possibility was the different distributions of connexin (Cx), the representative gap junction protein,^51^ between the hippocampus and neocortex. Although the high distribution of Cx expression is identical around astrocytic end-feet, the inner localization and structure of Cx within astrocytes are different^52^; Cx is heterogeneous in the hippocampus but homogeneous in the neocortex. Importantly, the suppression of astroglial Cx43, a major category of Cx expressed in astrocytes, can obstruct seizure development,^53^ supporting the pivotal role of astrocytes in seizure ignition.^54^ Testing whether the structural differences in astrocytes can affect the icDC appearance pattern would be fascinating to consider the underlying different mechanisms of MTLE and NE.

### icDCs and icHFOs versus epilepsy seizure outcome

We revealed that the resection of the core areas of icDCs, as well as icHFOs^23^ significantly correlated with favourable outcomes. Although no study has clearly shown this relationship, particularly with icDCs, Modur *et al*. have notably documented that the complete resection of electrodes showing icDCs and icHFOs preferably correlated with favourable outcomes.^18,55^ Notably, low-voltage fast activities (LVFA), well known to follow typical icDCs,^17,34,50,56^ have already been described to be associated with favourable outcomes.^57,58^ Therefore, our findings, as a multi-institutional study, can be interpreted as corresponding phenomena with a close correlation between them; the only differences are in the availability of icDCs due to the recording conditions. However, the relevance to favourable outcomes regarding HFOs remains controversial because a recent prospective study^59^ described the negative results and raised questions about previous positive results using interictal^60^ and ictal HFOs.^23^ This finding might prompt us to reconsider the significance by observing the diverse appearance patterns of interictal HFOs over time.^61^ We propose that the analysis of icDCs as well as icHFOs will further support the delineation of SOZ that should be resected to achieve a better prognosis.

Next, a notification should be addressed regarding the cases in which the core electrodes of either icDCs or icHFOs defined in this retrospective study were not completely resected in the surgery for the following reasons. For icDCs, seven patients achieved favourable outcomes without complete resection of core electrodes of icDCs, whereas eight patients showed poor outcomes without complete resection of icDCs (Fig. 7B, left). Among the former seven patients, tumour resection was mainly executed in three patients, ordinal anterotemporal lobectomy was executed in two patients because the core area of icDCs was in the posterior part of the temporal lobe, and the core electrode of icDCs was not completely resected because of language function in one patient. Among the latter eight patients, the core electrode of icDCs was not completely resected because of the eloquent cortex, such as the motor and visual areas. Only part of the resection of core electrodes of icDCs was executed because of anatomical reasons in two patients. Additionally, the electrodes were not considered the core electrodes of icDCs during presurgical evaluation in two patients because the amplitudes of icDCs were relatively small. Similarly, for icHFOs, four patients achieved favourable outcomes without complete resection of core electrodes of icHFOs, whereas seven patients showed poor outcomes without complete resection of icHFOs (Fig. 7B, right). Among the former four patients, three were the same patients above who achieved favourable outcomes without complete resection of icDCs; ordinal anterotemporal lobectomy was executed in one patient because the core area of icHFOs was in the posterior part of the temporal lobe. Among the latter seven patients, six were the same patients above who showed poor outcomes without complete resection of icDCs; ordinal anterotemporal lobectomy was executed in one patient because the core area of icHFOs was in an eloquent cortex.

Although our cohort study contains a selection bias in terms of intractable epilepsy patients, the relationships between the pathologies and outcomes favoured previous reports^62^; FCD Type IIa more often showed poor outcomes with frequent Engel IV than other pathologies. icHFOs were less likely to occur in specific pathologies (FCD Ib; 38%, FCD IIa; 49%), whereas a clear difference was not revealed regarding icDCs appearance among all pathologies. This finding suggests that complete resection of the core area of icDCs, rather than pathological differences, would be responsible for the favourable prognosis. Although limited reports have documented the pathological relevance to icDCs, our group recently successfully delineated the two types of icDCs, i.e. rapid development and slow development patterns, depending on waveform cluster analysis consistent with different pathology type.^63^ Further investigation with more detailed observations in specimens could reveal the pathological characteristics of icDCs.

### Future concerns and limitations

Mainly four future concerns and limitations should be noted as follows. First, the latency between icDCs and icHFOs was consistent and significant, but it may be relatively small, such as 2 s on average (Figs. 5 and 6). This difference may raise the negative concern that icDCs recorded here may not represent activity truly preceding icHFOs. However, the recording conditions for both icDCs and icHFOs were set to maximally record both; by contrast, icDC recording conditions may be less sensitive because we adopted an AC but not a DC amplifier. Furthermore, as shown in Table 3, the occurrence rate of icDCs was 86% as opposed to 65% for icHFOs, and those of icHFOs in FCD Ib and IIa were less than 50% (38 and 49%), whereas those of icDCs were 82 and 77%, respectively. Thus, icHFOs likely occur less frequently and later than icDCs. Future developments in recording methods may reveal the detailed timings of both activities, but the most important finding to note here is that both markers of icDCs and icHFOs, as currently measured, individually and significantly correlated with favourable postoperative outcomes. Some arguments may persist that our identified icDCs may be unstable activities caused by artefacts such as drift voltage because of the usage of platinum electrodes. Ag/AgCl electrodes have been reported to be most suitable for recording slow potential activities concerning the stability of the static membrane potential.^64^ Accordingly, we used Ag/AgCl electrodes for scalp EEG recordings but relatively stable platinum electrodes^65^ for intracranial recordings considering Ag toxicity to the brain. We have already documented the availability of several material electrodes with high input impedance to efficiently record the slow potentials.^66^ As mentioned in the Methods section, we only judged the slow potentials that were reproducible concerning their occurrence pattern, such as polarities and duration, to ensure the credibility of icDCs in a given electrode (the reproducibility of icDCs in different seizures in a given patient is clearly shown in Fig. 4). We can also exclude the possibility that they were spurious waveforms caused by volume conductions because icDCs were observed only at a limited number of electrodes without observations at adjacent electrodes.

Second, we excluded the patients showing long-lasting icDCs or icHFOs over 30 s for the duration analysis because of the fixed analysis time window of the software. Thus, it is possible that we may underestimate the duration of them. However, we consider that this methodological limitation has not affected the onset comparison between icDCs and icHFOs. It is because our main purpose of this paper is to compare the ictal onset activity of a particular focus between the icDCs and icHFOs.

Third, among the four patients excluded from the EEG analysis because of the lack of icDCs and icHFOs, three had a favourable outcome and one had a poor outcome. While it is noteworthy that one patient reported in previous study^5^ did not show conventional invasive EEG changes at all but showed icDCs and icHFOs, and the identification of the two biomarkers resulted in a favourable outcome. Thus, we consider that it is important to utilize all three EEG changes, i.e. three biomarkers, to identify the SOZ and achieve favourable outcome.

Fourth, we did not assess the importance of delayed slow frequency activities because they were not identified as core icDCs. Delayed slow potentials often occur subsequent to increasing the frequency or amplitudes of LVFA or repetitive spikes.^50^ These activities are not detected only in a restricted area but also sometimes over a wider area. However, this phenomenon does not contradict the significance of icDCs at seizure onset in the pathogenesis of seizure development. Given that LVFA onset seizures frequently propagate to the limbic and extralimbic areas^17,67^ including contralateral sites,^57^ it seems consistent that delayed-onset icDCs following LVFA or repetitive spikes are occasionally widely observed during the seizure. To distinguish between these two types of slow potentials, we described slow potentials at seizure onset as active DCs and slow potentials subsequent to an obvious increase in neuronal excitation (LVFA, repetitive spikes) observed during the seizures as passive DCs (the details have been described previously^68^). Interestingly, Zimmer *et al*. reported that as a consequence of ictal phenomena rather than ictal ignition processes, hypo/hypermetabolism in FDG-PET studies, in which we usually observe a wider anomaly than our prediction of the SOZ, can be attributed to glia-related function rather than neuronal function.^69^ This finding can be partly attributed to the widely observed delayed-onset passive DCs.

Here, we highlighted for the first time the positive relationships between postoperative outcome and icDCs at seizure onset. Our findings will encourage clinicians/researchers to focus more on the appropriate recording condition to detect icDCs to be validated in a much larger dataset for both animal and human studies. Thus, the extent to which electrodes with delayed icDCs may be resected is a practical issue to be addressed in the future. A prospective study considering icDCs at seizure onset in addition to the current procedure for determining the resection extent would be desirable for more advanced surgical treatment in the future, if clinically permitted.

## Conclusions

Based on the unified metric using multi-institute data, we verified that icDCs can appear within the SOZ more frequently than icHFOs and that icDCs significantly precede icHFOs both among and within the patients, particularly in NE patients. Notably, the present results can endorse the usefulness of icDCs to achieve favourable surgical outcome. We hope our findings can lead to additional therapeutic strategies in epilepsy surgery and contribute to an enhanced understanding of glial-neuron interactions considered to be related to the acquisition of epileptogenicity.

## Acknowledgements

We would like to thank the patients and their families involved in this study. I would also like to thank the doctors dedicated to the treatment of the patients.

## Supplementary Material

fcac222_Supplementary_Data

## Abbreviations

AC amplifier =: alternate current amplifier
AUC =: area under the curve
BDNF =: brain-derived neurotrophic factor
cSOZ =: conventionally defined seizure-onset zone
Cx =: connexin
FCD =: focal cortical dysplasia
FDG-PET =: ^18^F-fluorodeoxyglucose positron emission tomography
HS =: hippocampal sclerosis
ISA =: infraslow activity
LVFA =: low-voltage fast activities
MTLE =: mesial temporal lobe epilepsy
NE =: neocortical epilepsy
ROC curve =: receiver operating characteristic curve
SOZ =: seizure-onset zone
STFT =: short-term Fourier transformation
TC =: time constant
