# Supplementary material for: Ictal direct current shifts contribute to defining the core ictal focus in epilepsy surgery
Source: Brain Commun. 2022 Sep 3;4(5):fcac222. doi: 10.1093/braincomms/fcac222 (PMC9639799; doi:10.1093/braincomms/fcac222)
Supplement: fcac222_Supplementary_Data [file fcac222_Supplementary_Data.zip › Supplementary Table1.docx]

**Supplementary Table 1: Summary of the clinical examinations of the newly enrolled patients (the remaining 16 patients were described previously^4^)**

| **Patient #** | **Sex** | **Age at onset** | **Diagnosis** | **Age at surgery** | **MRI** | **FDG-PET** | **Electrode placement** | **Pathology** | **outcome** |
| --- | --- | --- | --- | --- | --- | --- | --- | --- | --- |
| 1 | F | 19 | L MTLE | 41 | L HS | L TP↓ | L F, T, P | FCD IA | Ic |
| 2 | M | 43 | L LTLE | 52 | L T | L T↓ | L F, T, P, O | arteriovenous malformation/gliosis | IId |
| 3 | F | 9 | R PLE | 27 | R PO | R P↓ | R P, O | FCD IIB | IVa |
| 4 | M | 15 | L FLE | 45 | no lesion | N.S. | L F, P | FCD IA | IIIa |
| 5 | M | 28 | L PLE | 61 | L P | L TP↓ | L T, P | Oligoastrocytoma | Ic |
| 6 | F | 23 | R MTLE | 30 | R T | R T↓ | R T, P, O | FCD IIA | IVa |
| 7 | F | 12 | L MTLE | 28 | L HS | L T↓ | L F, T, P | non-neoplastic brain tissue | IVa |
| 8 | M | 6 | R MTLE | 39 | R HS | R T↓ | R F, T, P | HS (+ FCD IA) | Ia |
| 9 | M | 18 | R FLE | 29 | no lesion | N.S. | R F, P | FCD IA | IIIa |
| 10 | M | 27 | L MTLE | 41 | L HS | L T↓ | L F, T, P | HS (+ FCD IA) | Ia |
| 11 | M | 1 | R FLE | 17 | no lesion | R F↓ | R F, T, P | FCD IA | IVa |
| 12 | F | 13 | L FLE | 16 | L F | L F↓ | L F, T, P | DNT | Ia |
| 13 | M | 25 | L FLE | 55 | no lesion | L F↓ | L F, T, P | Perivascular glial satellitosis  in the white matter | Ia |
| 14 | M | 4 | L MTLE | 11 | no lesion | L T↓ | L F, T, P | gliosis | Ia |
| 15 | M | 6 | R FLE | 18 | no lesion | R op, Ins↓ | R F, T, P | mild gliosis | IIa |
| 16 | F | 7 | R MTLE | 52 | no lesion | N.S. | LT and R F, T, P | HS | Ia |
| 17 | F | 13 | L MTLE | 23 | no lesion | bilateral T↓ | L F, T, P | gliosis | Ia |
| 18 | M | 10 | R MTLE | 25 | HS (R<L) | R T↓ | bilateral T | no resection | IIa |
| 19 | M | 11 | R FLE | 13 | no lesion | N.A. | R F, T, P | FCD IIA | IIIb |
| 20 | F | 12 | R LTLE | 30 | R T nodular heterotopia, HS | N.A. | R T P O | Nodular heterotopia, polymicrogyria | Ia |
| 21 | F | 0 | L FLE | 27 | L C abnormal sulcus | N.A. | L F, P | FCD IIA | IIIa |
| 22 | F | 4 | R FLE | 24 | R F bottom of sulcus | N.A. | R F, T, P | FCD IIB | Ia |
| 23 | M | 3 | R LTLE | 17 | no lesion | R STG↓ | R F, T | non specific | IIb |
| 24 | M | 10 | R FLE | 28 | R op, Ins | N. A | R F | FCD IA | IVa |
| 25 | F | 5 | R FLE | 21 | no lesion | R T↓ | R F, T, P | non specific | IVa |
| 26 | M | 6 | R FLE | 24 | R F | R F↓ | R F | FCD IIB | Ia |
| 27 | F | 3 | L MTLE | 46 | bilateral HS (L>R) | L T↓ | bilateral T, P, O | HS (classical, dispersion) | IVa |
| 28 | M | 9 | L MTLE | 29 | L HS | L F, T↓ | L F, T | HS (+ gliosis) | Ia |
| 29 | M | 21 | MTLE | 26 | no lesion | R T↓ | bilateral T, P, O | no resection | − |
| 30 | M | 7 | L OLE | 15 | L O | L O↓ | L T, P, O | gliosis | Ia |
| 31 | M | 3 | L FLE | 12 | L op | L op↓ | L F, T, P | FCD IIA | IVa |
| 32 | F | 11 | R LTLE | 24 | R T | R TP↓ | R F, T, P | FCD IA (heterotopia) | IVa |
| 33 | M | 4 | L FLE | 49 | L F | L F, T↓ | R T and L F, T, P | FCD IIB | Ia |
| 34 | M | 6 | R FLE | 18 | R SFG | R SFG↓ | R F, P | FCD IIB | IIIa |
| 35 | M | 20 | L FLE | 40 | no lesion | L F↓ | L F, P | FCD IIA | IVa |
| 36 | M | 8 | L LTLE | 35 | L T | L T↓ | bilateral T | non specific | IId |
| 37 | M | 5 | R FLE | 16 | R F | N.S. | R F, P | FCD IIB | Ib |
| 38 | F | 26 | R LTLE | 49 | no lesion | R T↓ | bilateral T | no resection | − |
| 39 | F | 15 | L LTLE | 19 | L T | L T↓ | L F, T, P | no resection | − |
| 40 | F | 11 | R MTLE | 15 | R T | R T↓ | R F, T, P | FCD IA | Ia |
| 41 | M | 13 | L PTLE | 23 | no lesion | L TO↓ | L F, T, P, O | no resection | − |
| 42 | F | 3 | R FLE | 5 | R F | R F↓ | R F, P | FCD IIA | Ib |
| 43 | M | 2 | R FLE | 30 | no lesion | R F↓ | R F | non specific | Ia |
| 44 | F | 3 | R LTLE | 15 | R pT | R pT↓ | R T | FCD IB | Ia |
| 45 | M | 6 | R FLE | 28 | R T | R T↓ | R F, T, P | FCD IIB | Ia |

FLE: frontal lobe epilepsy, MTLE: mesial temporal lobe epilepsy, LTLE: lateral temporal lobe epilepsy, PTLE: parietal lobe epilepsy

SFG: supra frontal gyrus, STG: supra temporal gyrus, op: operculum, Ins: insular, TP: temporo-parietal, pT: posterior temporal, TO: temporo-occipital

HS: Hippocampal sclerosis, FCD: Focal cortical dysplasia, DNT: dysembryoplastic neuroepithelial tumor

N.A.: not acquired, N.S.: not significant
