# Supplementary material for: Ictal direct current shifts contribute to defining the core ictal focus in epilepsy surgery
Source: Brain Commun. 2022 Sep 3;4(5):fcac222. doi: 10.1093/braincomms/fcac222 (PMC9639799; doi:10.1093/braincomms/fcac222)
Supplement: fcac222_Supplementary_Data [file fcac222_Supplementary_Data.zip › Supplementary figure.pdf]

<Supplementary Figure>

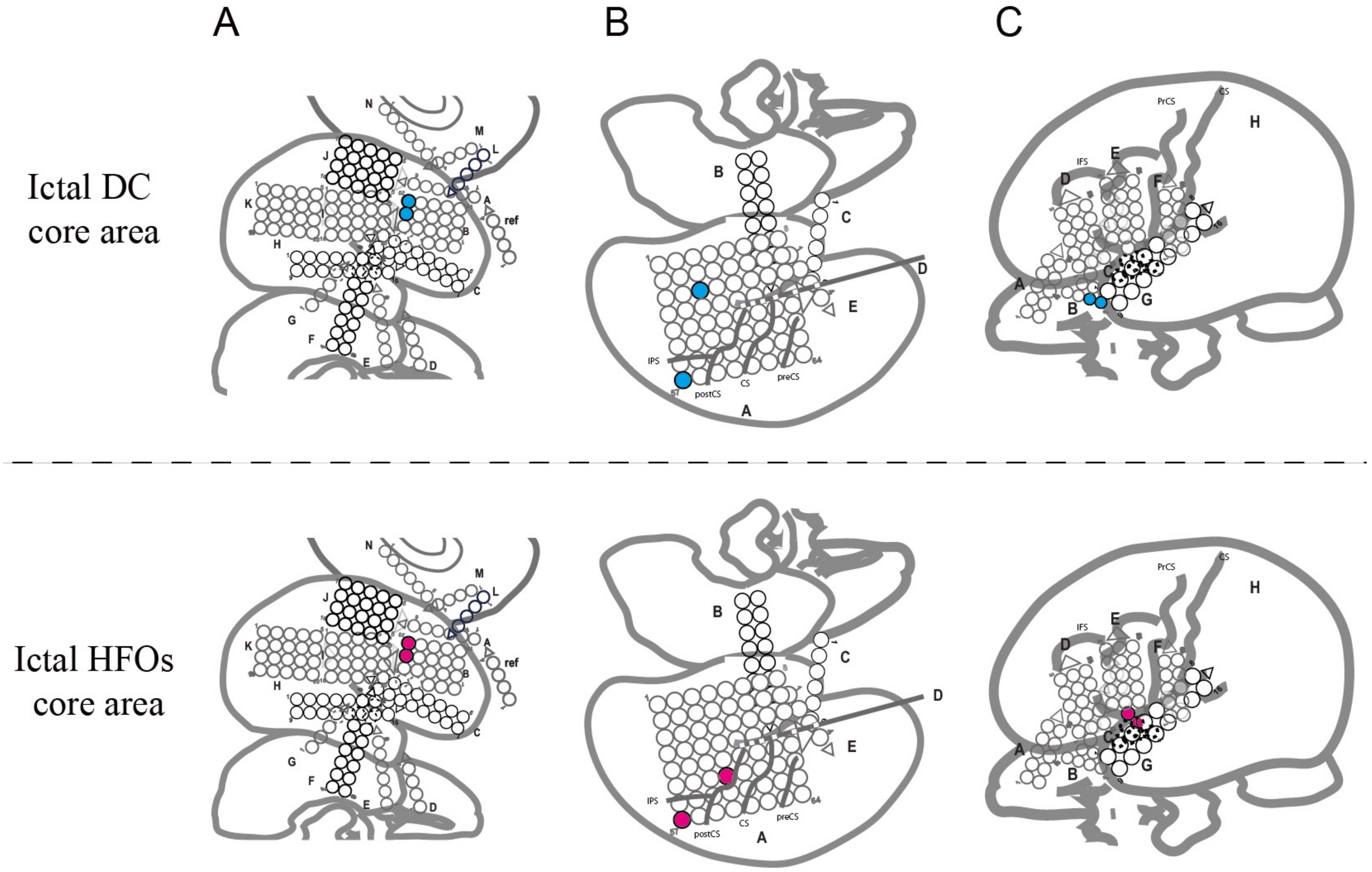

Supplementary Figure 1 Different concordance rates of the core electrodes of icDCs and icHFOs
(A) A 17-year-old male right frontal lobe epilepsy patient.
(B) A 32-year-old female left temporal lobe epilepsy patient.
(C) A 24-year-old male left frontal lobe epilepsy patient.
The location of the implanted electrodes in each patient is described within the brain schema.
The two defined core electrodes of icDCs (blue) and icHFOs (red) in the representative patients are independently illustrated.
Completely matched (A), partially matched (B), and unmatched (C) cases were observed at the electrode level.
